# Kyste hydatique du muscle psoas: à propos d’un cas

**DOI:** 10.11604/pamj.2016.24.302.10098

**Published:** 2016-08-09

**Authors:** Imane Alaoui, Fatimazahra Hjoui, Meriem Doumbia, Sarra Aoufi, Mohammed Lyagoubi

**Affiliations:** 1 Laboratoire de Parasitologie-mycologie, Centre Hospitalier Universitaire IBN SINA, Faculté de Médecine et de Pharmacie de Rabat, Université Mohammed V, Maroc

**Keywords:** Kyste hydatique, psoas, sérologie hydatique, Hydatid cyst, psoas, serology hydatid

## Abstract

Le muscle psoas est une localisation exceptionnelle du kyste hydatique. Le but de cette lettre est de rapporter l'observation d'un kyste hydatique localisé au niveau du muscle psoas gauche chez un patient âgé de 32 ans. L'échographie, la tomodensitométrie ainsi qu'une sérologie hydatique positive ont contribué au diagnostic préopératoire. Le patient a été opéré par incision para rectale gauche type Jalaguier. L'examen microscopique du culot de centrifugation du liquide hydatique a mis en évidence des scolex et de nombreux crochets confirmant ainsi le diagnostic. L'évolution était bonne sans récidive après le traitement chirurgical.

## Abstract (en)

The psoas muscle is an uncommon location for hydatid cyst. Our study reports the case of a 32-year-old patient with hydatid cyst located at the left psoas muscle. Ultrasound, CT scan as well as a positive hydatid serology contributed to the preoperative diagnosis. The patient underwent left pararectal incision (Jalaguier). Microscopic examination of the hydatid liquid pellet highlighted scolices and many hooks confirming the diagnosis. The evolution was good with no recurrence after surgical treatment.

## Introduction

Le kyste hydatique ou hydatidose est une anthropozoonose due au développement chez l'homme de la forme larvaire du taenia *Echinococcus granulosis*. Il sévit à l'état endémique et constitue un véritable problème de santé publique au Maroc. La localisation musculaire isolée est une entité inhabituelle même dans les pays endémiques. Sa fréquence serait de 2 à 3% de toutes les localisations. Le psoas reste une localisation exceptionnelle. Dans la littérature mondiale quelques cas cliniques du kyste hydatique du muscle psoas ont été décrits [**1**]. Nous rapportons une nouvelle observation d'un kyste hydatique localisé au niveau du muscle psoas.

## Patient et observation

**Figure 1:**
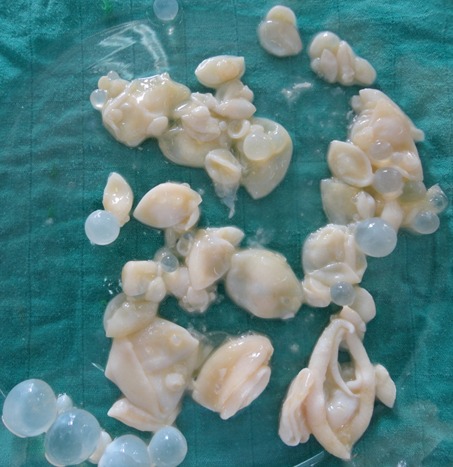
Membrane proligère et vésicules filles du kyste hydatique

**Figure 2:**
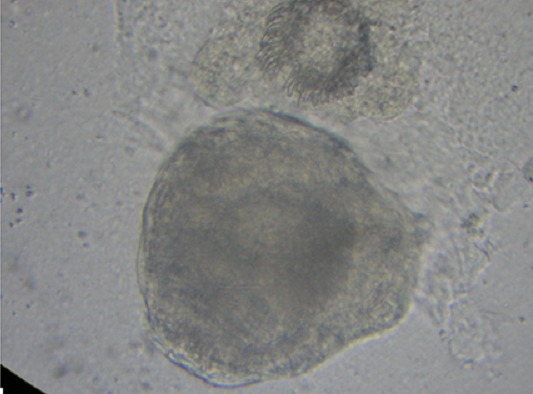
*Echinococcus granulosis*: scolex et crochets en couronne

Mr K.A âgé de 32 ans, sans antécédents pathologiques particuliers, se plaignait depuis quatre mois des douleurs diffuses du flanc gauche avec sensation de pesanteurs à ce niveau associées à une fièvre sans amaigrissement ni altération de l'état général. L'examen clinique trouvait un patient fébrile à 38,7°C, avec une masse au niveau de la fosse iliaque gauche, sensible à la palpation et fixée au plan profond. L'échographie abdominale avait mis en évidence une image anéchogéne multiloculaire cloisonnée, située au niveau de la fosse iliaque gauche. Le scanner a confirmé la présence d'un kyste hydatique stade III au dépend du muscle psoas gauche. Le reste de l'exploration ne montrait aucune autre localisation hydatique. La sérologie hydatique par test ELISA était positive à 24 UI/ml et la réaction d'immunofluorescence indirecte à 80 UI/ml. Le patient a été opéré par une incision para rectale gauche type Jalaguier découvrant un kyste hydatique unique retropéritonéal localisé sur la face antérieure du muscle psoas gauche, constitué de plusieurs logettes séparées du septas. Dans notre laboratoire, nous avons reçu la pièce d'exérèse chirurgicale constituée d'une membrane proligère du kyste hydatique et de nombreuses vésicules filles (Figure 1). L'examen microscopique du culot de centrifugation du liquide hydatique a mis en évidence des scolex et de nombreux crochets (Figure 2) confirmant ainsi le diagnostic.

## Discussion

L'hydatidose est une anthropozonose due à la forme larvaire d´Echinococcus granulosus, un petit ténia parasitant, à l´état adulte, le tube digestif du chien qui constitue l'hôte définitif. L'infestation du chien se fait par voie digestive et serait secondaire à la consommation de viscères parasités, notamment le foie et les poumons de l'hôte intermédiaire: le mouton. Ce dernier, constituant le principal réservoir du ténia échinococcus, se contamine en broutant l'herbe souillée par les déjections du chien contenant les oeufs du parasite. L'homme n'est qu'un hôte intermédiaire accidentel, il s'infecte soit directement au contact du chien parasité soit indirectement par une ingestion d'aliments souillés. L'homme constitue une impasse épidémiologique [2]. Toutes les localisations de l'hydatidose ont été décrites, dans 90% des cas, elle touche le foie et le poumon. Cette distribution s'explique par la dissémination sanguine du parasite et les flux sanguins de la circulation portale. Les oeufs du ténia échinocoque ingérés par l'homme libèrent dans l'intestin l'embryohexacante. Ce dernier franchit la muqueuse intestinale et passe dans la circulation porte. Sa taille et sa plasticité lui permettent de passer partout. Le courant portal emporte cet embryon vers le premier barrage qui est le foie où il s'arrête six fois sur dix, sinon par l'intermédiaire des veines sus-hépatiques, le parasite gagne la veine cave inférieure, le cœur droit, puis le poumon qui constitue le deuxième barrage où il est retenu trois fois sur dix. Une fois sur dix l'embryohexacante franchit les deux barrages, se retrouve dans la grande circulation et peut se loger dans n'importe quel endroit de l'organisme. L'atteinte des tissus mous est inhabituelle, elle est décrite dans 0,5 à 4,7% des cas et intéresse principalement les muscles squelettiques du cou et des membres inférieurs. Celle-ci est due d'une part au degré de la vascularisation des tissus et aux contractions musculaires qui empêcheraient le développement de la larve et d'autre part à la richesse en acide lactique du muscle qui empêcherait la croissance de l'hydatide [3]. Le diagnostic de kyste hydatique du muscle psoas, souvent difficile, est celui d'une masse abdominale, à localisation iliaque ou lombaire, rénitente, fixée au plan profond. Certains kystes peuvent être révélés par des complications à type de compression nerveuse, urinaire, vasculaire, ou par une surinfection par voie hématogène pouvant engendrer un sepsis parfois sévère [4]. L'imagerie est essentielle au diagnostic et au bilan pré thérapeutique. L'échographie est un examen anodin de première intention avec une fiabilité diagnostique estimée à 96%. L'aspect échographique reproduit les stades de la classification de Gharbi et traduit le stade évolutif de la maladie [5]. Dans les localisations profondes comme le psoas l'intérêt d'une étude scanographique est nécessaire. Le scanner permet un diagnostic facile avec un bilan topographique plus précis. Le bilan biologique apporte une certaine finesse au diagnostic de l'hydatidose surtout en cas de problème diagnostique et garde un intérêt majeur dans le cadre des enquêtes séro-épidémiologiques et du suivi après traitement. L'hyper éosinophilie est inconstante et n'a d'intérêt que dans l'orientation du diagnostic. La biologie se résume essentiellement à la sérologie hydatique. Elle est d'un grand apport diagnostic lorsqu'elle est positive. Sa négativité n'élimine pas le diagnostic de kyste hydatique d'où l'obligation d'une confrontation entre la clinique, l'imagerie et la biologie. Notre observation illustre l'intérêt de la sérologie hydatique dans le diagnostic positif de cette parasitose, toutefois, il existe une proportion non négligeable de faux négatifs, variable en fonction de la localisation du kyste [3]. Pour améliorer le rapport sensibilité/spécificité, la plupart des auteurs préfèrent associer 2 techniques sérologiques, une quantitative: hémaglutination indirecte, immunofluorescence, ELISA et l'autre qualitative: immunoélectrophorèse, électrosynérèse [6]. Outre son rôle dans la confirmation diagnostique, la sérologie hydatique permet de suivre l'évolution post thérapeutique du kyste hydatique, de formuler un pronostic et de dépister précocement une hydatidose secondaire. Ainsi, toute élévation dans le semestre qui suit l'intervention est synonyme de récidives ou de localisations hydatiques passées inaperçues. Les méthodes qualitatives et quantitatives sont d'interprétation difficile, néanmoins, le Western Blot et l'immunoempreinte sont plus sensible et plus spécifique [7]. Le seul traitement curatif du kyste hydatique du psoas est chirurgical. Le traitement médical à base d'albendazole reste destiné aux malades inopérables ou en cas de récidive massive en complément de la chirurgie. L'abord chirurgical extra péritonéal est préférable pour éviter l'ouverture de la cavité péritonéale et ainsi éliminer tout risque de dissémination hydatique intra péritonéale [8].

## Conclusion

Le kyste hydatique isolé du psoas reste une entité rare. Le diagnostic repose essentiellement sur L´échographie et le scanner, la biologie apporte des éléments supplémentaires. Le meilleur traitement repose essentiellement sur la prévention de l'hydatidose qui malheureusement continue à sévir à l'état endémique dans notre pays et représente un véritable fléau social.
